# Differential Joint-Specific Corticospinal Tract Projections within the Cervical Enlargement

**DOI:** 10.1371/journal.pone.0074454

**Published:** 2013-09-18

**Authors:** Curtis O. Asante, John H. Martin

**Affiliations:** 1 Department of Physiology, Pharmacology, and Neuroscience, City College of the City University of New York, New York, New York, United States of America; 2 Department of Neuroscience, Columbia University, New York, New York, United States of America; University of Bordeaux, France

## Abstract

The motor cortex represents muscle and joint control and projects to spinal cord interneurons and–in many primates, including humans–motoneurons, via the corticospinal tract (CST). To examine these spinal CST anatomical mechanisms, we determined if motor cortex sites controlling individual forelimb joints project differentially to distinct cervical spinal cord territories, defined regionally and by the locations of putative last-order interneurons that were transneuronally labeled by intramuscular injection of pseudorabies virus. Motor cortex joint-specific sites were identified using intracortical-microstimulation. CST segmental termination fields from joint-specific sites, determined using anterograde tracers, comprised a high density core of terminations that was consistent between animals and a surrounding lower density projection that was more variable. Core terminations from shoulder, elbow, and wrist control sites overlapped in the medial dorsal horn and intermediate zone at C5/C6 but were separated at C7/C8. Shoulder sites preferentially terminated dorsally, in the dorsal horn; wrist/digit sites, more ventrally in the intermediate zone; and elbow sites, medially in the dorsal horn and intermediate zone. Pseudorabies virus injected in shoulder, elbow, or wrist muscles labeled overlapping populations of predominantly muscle-specific putative premotor interneurons, at a survival time for disynaptic transfer from muscle. At C5/C6, CST core projections from all joint zones were located medial to regions of densely labeled last-order interneurons, irrespective of injected muscle. At C7/C8 wrist CST core projections overlapped the densest interneuron territory, which was located in the lateral intermediate zone. In contrast, elbow CST core projections were located medial to the densest interneuron territories, and shoulder CST core projections were located dorsally and only partially overlapped the densest interneuron territory. Our findings show a surprising fractionation of CST terminations in the caudal cervical enlargement that may be organized to engage different spinal premotor circuits for distal and proximal joint control.

## Introduction

The corticospinal tract (CST) provides a direct path for the motor cortex to spinal motor circuits. From the earliest studies, we learned of the predominance of the motor cortex muscle and joint representation; the familiar homunculus in humans [1]. This has been reinforced using intracortical microstimulation (ICMS) for fine-grained mapping of the motor cortex in animals [2]. However, many aspects about the basic organization of motor cortex control over limb muscles and joints via the CST are not known. Indeed, many studies point not to a simple representation of muscles or joints, but to more complex and integrative control [3], [4].

The CST projects to the spinal cord to contact motoneurons in select species and interneurons, in all species [5]. Possible functions of corticomotoneuronal cells [6], [7] in individuated muscle control can be inferred by their direct connections. While we have an understanding of the diverse physiological actions of the CST on spinal interneuronal systems [8]–[10], and a growing inventory of CST-to-interneuron projections [11], we have little insight into the possible functional organization of these connections.

In this study, we focused on the anatomical organization of motor cortex joint control at the spinal level. As sites in motor cortex preferentially represent different forelimb joints, it follows that during tasks that recruit the CST, the cortical representations of different forelimb joints [12] may each comprise parallel CST paths for controlling separate joints. We addressed two questions. First, is there an anatomical substrate for CST joint control, whereby cortical sites controlling particular joints have differential cervical segmental terminations? Alternatively, does CST joint control emerge from segmentally undifferentiated terminations? Second, since important motor actions of the CST are expressed via segmental interneurons, is there a topographic relationship between CST projections from the different cortical joint representations and spinal premotor interneurons [13], [14] associated with muscles that act around the corresponding joints?

We focused on the forelimb motor cortex representation and the cervical enlargement segments that are the substrates for forelimb control. We studied this in the mouse because we could identify putative spinal premotor interneurons using retrograde transneuronal transport of pseudorabies virus (PRV) in mature mice and because a functional neuroanatomical study in that species could inform molecular genetic studies of cortical projection and spinal interneurons [15], [16]. We determined the motor cortex motor representation using ICMS and mapped connections from identified forelimb joint-specific sites using anterograde tracers. We used intramuscular injection of two PRV strains–at a survival time for disynaptic transfer from muscle–to identify putative last-order interneurons. This approach has been used recently to identify last-order interneurons in neonatal mouse lumbar spinal cord [17]. Our findings show fractionation of CST connections within the cervical enlargement. As joints are represented in motor cortex, there appears to be a complementary spinal representation that provides CST projections from joint-specific sites access to distinctive spinal territories. For distal control, our findings point to a CST projection organized to engage spinal premotor circuits preferentially.

## Methods

### Ethics Statement

Experiments were conducted on adult male and female C57/BL6 mice. All procedures were approved by the Institutional Animal Care and Use Committees of City College of the City University of New York, New York State Psychiatric Institute, and Columbia University.

### Intracortical Microstimulation

For ICMS motor mapping, anesthesia was induced with a ketamine/xylazine mixture (100/10 mg/kg; IP) and maintained using IP ketamine injections to render the animal unresponsive to paw pinch while maintaining muscle tone. Animals were placed in a stereotaxic frame. Body temperature was maintained at 39°C by a heating pad. A craniotomy was made over the forelimb area of M1. We used tungsten microelectrodes (Microprobe, Inc.; 0.5 MOhm impedance; 0.081 mm shaft diameter, 1–2 µm tip diameter). Electrode penetrations were made perpendicular to the pial surface and approximately 0.3 mm apart. In all animals, the region sampled was the same, from 1 to 1.9 mm lateral to bregma and up to 2.1 mm rostral to bregma. Motor effects produced by microstimulation occurred at the lowest stimulus current when the electrode was at the depth that we recorded multiunit activity with the largest amplitude spikes (typically 0.8–1.0 mm below the pial surface); this was presumably laver V.

Stimuli (45 ms duration train, 330 Hz, 0.2 ms biphasic; every 2 sec) were delivered using an isolated pulse stimulator in constant current stimulation mode (A–M Systems). The threshold was defined as the lowest current that consistently produced a motor effect on >50% of trials. For a given site, we started at a low current and first determined the threshold for evoking a contralateral response. The thresholds were then examined in reverse through the loss of the responses with decreasing currents. We randomized placement of the electrode to prevent biasing our results by anesthesia level or other state-dependent changes. Stimulation currents were up to 100 µA. For each penetration, the type of motor effect produced by a threshold stimulus was determined on the basis of the evoked phasic kinematic change; adjacent joints were stabilized. Limb posture was the same for all experiments; with the shoulder and elbow extended, and the wrist plantar flexed.

### Electromyography

We recorded electromyographic (EMG) responses from forelimb muscles using percutaneous Ni-chrome wire electrodes and a differential AC amplifier with low and high pass filtration (A–M Systems). EMG recording wires were deinsulated at the tip (1 mm), a small hook was formed by bending the wire over the needle tip, and inserted into muscle using a 26-gauge hypodermic needle. Because of the hook at the end of the EMG lead, when the needle was withdrawn the wire remained securely embedded within the muscle. We recorded differentially, with two wire electrodes within each muscle. EMG signals were acquired using an analog-to-digital converter (Digidata; Axon Instruments) at 20 kHz per channel and processed using the program AxoGraph for the Apple Macintosh computer. For analysis and display, EMGs were first rectified and then averaged.

### Iontophoretic Application of Tracers

Immediately after ICMS, in selected experiments we injected biotinylated dextran amine (BDA, Invitrogen; 10% in 0.1 M phosphate buffer (PB), Lucifer yellow dextran amine (LY-DA; Invitrogen; in 0.1 M PB) or dextran alexa fluor (DAF; Invitrogen; 10% in 0.1 M PB) into specific sites of the forelimb area of motor cortex to anterogradely label joint-specific CST projections. Injections were made using glass micropipettes (15–20 µm tip diameter). The current for iontophoresis was set to 7 µA for 5 min in alternating mode (7 s on, 7 s off; Midgard™ precision current source; Stoelting Co.). The average injection site diameter was 279±11µM (n = 3 mice). For each animal, we injected BDA with either LY-DA or DAF at sites separated by at least 400 µm. After a minimum and maximum post surgery survival of 14 and 21 d respectively, mice were given an anesthetic overdose and perfused through the heart with heparinized (0.1%) saline followed by 4% paraformaldehyde. The brain and spinal cord were removed, post-fixed in the same fixative at room temperature for 2 hr, and transferred to 20% sucrose in 0.1 M PB at 4°C overnight. Transverse sections of the cervical enlargement (C5 to C8; identified by counting roots) were cut at 40 µm and processed for tracer histochemistry. For visualization of BDA, sections were incubated with ExtrAvidin cyanine 3 (Cy3; 1∶1000 to 1∶2000; Sigma) overnight at 4°C. For visualization of LY-DA and DAF, sections were incubated at 4°C overnight in PBS containing rabbit anti-LY-DA antibody (1∶1000; Invitrogen) or rabbit anti-alexa fluor antibody (1∶400; Molecular Probes) in blocking buffer (3% donkey serum in 1×PBS with 0.2% Tween 20, pH 7.4). After rinsing, sections were incubated for 2 hr at room temperature (RT) in blocking buffer containing 0.2% anti-rabbit secondary antibody conjugated to FITC (1∶500; pH 7.4).

### Analysis of Topography of CST Terminations

**Figure 1:**
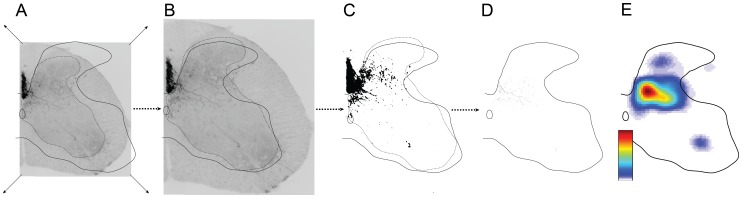
Methods used for generating color-coded density heat maps. To illustrate the method, we show a single spinal cord section and, from left to right, the process of resizing the image to a standardized size (A to B), thresholding and skeletonization of the image with ImageJ (C to D) and converting to a color-coded heat map with Matlab (E). Color scale represents the number of pixels per square mm.

We developed a quantitative method for determining the topographic distribution of label within the gray matter in the cervical enlargement. Figure 1 shows the basic method for a single section. For each transverse section of the spinal cord, we captured images at 100 X using the MosiacJ plugin application for ImageJ (NIH) to create a montage. For the purposes of comparing across animals, the gray matter border and contours for individual sections were grouped using a graphics program (Adobe Photoshop and Adobe Illustrator, Adobe Systems) and the size of the gray matter, and associated contours, were normalized to standard dorsoventral and mediolateral lengths (A,B). Next, we simultaneously altered the threshold for all regions of interest (regions containing labeled CST axons) such that only CST axons were visible and background noise was at a minimum (C). To complete this task accurately and reliably, the threshold was always adjusted in full view of the original section, so that false-positives were not introduced. These images were then transferred back to Image J, where they were digitized using the skeletonize function (D) to erode all CST axons to a single pixel width. This results in images that represent CST axon density not CST axon thickness. The total number of pixels in a given area thus corresponds to the overall density of CST label in a given area of the section. Although shown in E for a single section, multiple pixelated images were grouped and further processed in Matlab (Mathworks) to generate regional density maps (i.e. heat maps) for each group (E). To ensure accurate registration between a heatmap and gray matter border within and across animals we used the base of the dorsal column as a fiduciary mark and constructed an average gray matter border in Matlab. A contour finding routine was written in Matlab and applied to the regional distribution map, where the density of CST axons was equal to or more than either 10% (corresponding to light blue through saturated red) or 60% (yellow/orange through saturated red). We counted spinal roots to determine the segmental level for tissue processing. Data were combined across the C5 and C6 segments and across the C7 and C8 segments. For each animal, at least 3 sections through each of the two levels were analyzed, converted and averaged.

For quantitative assessment of CST labeling we measured the amount of label within 4 regions of interest (ROI) in the contralateral gray matter: portion of laminae 3–4, located at the level of the “notch” on the lateral dorsal horn; laminae 4–5, to capture the region of dense labeling in between adjoining ROIs; lamina 6, at the base of the dorsal column; and a portion of laminae 7–9, just ventral to the ventral commissure.

### Retrograde and Transganglionic Labeling using Cholera Toxin b Subunit

Cholera toxin b subunit (CTb) was used to retrogradely label the motor pools of selected forelimb muscles and to anterogradely (i.e., transganglionically) label muscle afferents as a means to help localize CST terminations. CTb preferentially labels myelinated afferents [18], [19]. Studies have shown a correspondence between CTb-labeled proprioceptive afferents and intra-axonally labeled group 1 afferents [20] and group 1 field potentials [21]. We performed multiple injections of CTb (1∶1000, List Laboratories) unilaterally into the deltoid, elbow flexor, and the wrist extensor compartments (4×2.5 µL; 33 ga. Hamilton syringe). Our intent was to label selectively muscles that act at the same joint. For the wrist, we injected the extensor compartment, targeting predominantly Extensor Carpi Radialis (ECR; both long and short heads). For the elbow, we injected biceps (long and short heads). For the shoulder, we injected the Deltoid muscle group (spino- and acromiodeltoidus). We have optimized the CTb injections to maximally label the motoneurons of injected muscles, taking advantage of recent findings that many forelimb motor pools have an extended rostrocaudal distribution [22].We inserted the Hamilton syringe into the muscle, advanced it along the long axis of the muscle, and injected CTb as the needle was withdrawn. As needed to maximally label the muscle, we often re-advanced the needle and injected more tracer. We verified that there was no leakage of CTb. CTb was tinted with the dye Evans blue. After removal of the needle any residual tracer was blotted with a cotton swab and washed with saline. After 5 days, mice were deeply anesthetized and perfused as above. The spinal cord was dissected, post-fixed for 2 hours and then transferred to 20% sucrose in buffered saline (pH 7.4). Frozen sections (40 µm) were cut serially and collected in a 0.1 M PBS solution (pH 7.4). All tissue was cut in the coronal plane into 40µm sections. All sections were collected. For visualization of CTb, we incubated the sections in PBS containing goat anti-CTb (1∶2000; List Laboratories) at 4°C overnight. After rinsing, sections were incubated for 1 hour at RT in blocking buffer (3% donkey serum in 1×PBS with 0.2% Tween 20, pH 7.4). The sections were then incubated with donkey anti-goat conjugated to Cy3 or FITC (1∶800 and 1∶500 respectively; Millipore) for 2 h at RT. All sections were mounted on gelatin-coated slides, air-dried and coverslipped with Vectashield (Vector Laboratories). Transganglionic labeling was assessed using the same steps that were taken to determine CST topography (see above).

### Retrograde Transneuronal Tracing using Pseudorabies Virus

Like CTb, injections of pseudorabies virus (PRV) were also made unilaterally into the deltoid, biceps, the wrist extensor compartment (4×2.5 µL). We used two types of the Bartha strains–152, expressing green fluorescent protein (titer of either 2.72×10^8^ or 4.02×10^8^ pfu/ml) [23] and 614, expressing red fluorescent protein (titer of 1.86×10^8^ pfu/ml) [24]. This permitted labeling of two muscles in the same animal. Across experiments, each muscle was labeled with both strains. PRV was generously provided by Dr. Lynn Enquist (Princeton University, Princeton, NJ). After the appropriate survival time (see Results), mice were deeply anesthetized, perfused, and spinal tissue removed as described above. All tissue was cut in the coronal plane into 40µm sections. All sections were collected.

To visualize PRV-labeled spinal neurons, we double immunostained the sections with antibodies to green and red fluorescence protein (rabbit anti-GFP antibody, Invitrogen and rabbit anti-RFP antibody, Abcam). After rinsing sections in 1×PBS, sections were then incubated for 1 hour at RT in blocking buffer (3% donkey serum in 1×PBS with 0.2% Tween 20, pH 7.4). The sections were next incubated at 4°C overnight in PBS containing primary anti- GFP antibody (1∶1000) After rinsing with 1×PBS, sections were incubated for 2 hr at RT with FITC-conjugated donkey anti-rabbit antibody (1∶500; Jackson Immunoresearch Laboratories). Sections were then rinsed again in 1×PBS and incubated at 4°C overnight in PBS containing primary anti-RFP antibody (1∶1000). After rinsing with 1×PBS, sections were incubated for 2 hr at RT with donkey anti-goat conjugated to Cy3 (1∶800). All sections were mounted on gelatin-coated slides, air-dried and coverslipped with Vectashield (Vector Laboratories). For PRV-ChAT double immunostaining, the same double-staining process as above was repeated using anti-GFP to label PRV-infected interneurons. Sections were then immunostained for ChAT using goat anti-ChAT (1∶100; Millipore) followed by donkey anti-goat conjugated to Cy3 (1∶800).

To quantify PRV labeling, we randomly selected and processed (as above) sections through the cervical enlargement of the spinal cord. Using Neurolucida (Microbrightfield), we counted all labeled neurons on at least 4 sections per animal for combined C5/C6 levels and C7/C8 levels. The images of the counted sections were processed in Matlab to produce regional density maps (see above). All sections sampled were of similar sizes therefore removing the need to re-size the images as was the case when determining CST topography.

### Confocal Microscopy

To determine whether CST axons contacted spinal cord interneurons directly and to image PRV-ChAT double-labeled interneurons, we used laser-scanning confocal microscopy (LSM META510 and 710; Carl Zeiss) using two different fluorescent markers: FITC (488 nm excitation, 520 nm emission) and Cy3/rhodamine (543 nm excitation, 573 nm emission). To adjust color balance, contrast, and brightness in the confocal images, ImageJ (NIH) and Adobe Photoshop (Adobe Systems) were used. When comparing images, all capture and adjustment parameters were kept identical.

### Statistical Analyses

Standard statistical tests including student’s t-test and analysis of variance (ANOVA) were conducted using Microsoft Excel (Microsoft), Prism 4 (Graph Pad), and Statview. We used the Fisher’s PLSD post hoc test for repeated measures ANOVA.

## Results

We investigated the functional organization and spinal connectivity of the forelimb area of the motor cortex in adult mice, which includes the caudal and rostral forelimb areas [25], [26]. We used ICMS to map the representations of contralateral forelimb joints (shoulder, elbow, and wrist/digits) in order to target anterograde axon tracer injections for mapping the CST spinal terminations of identified joint-specific zones. Using image analysis, we analyzed the laminar distribution of CST terminations within the segmental levels of motoneurons supplying muscles for each joint. We compared the distributions of the joint-specific CST terminations with the locations of proprioceptive afferents using CTb and putative last-order interneurons, using retrograde transneuronal labeling of PRV in adult mice at a survival time that ensured transport across only one spinal cord synapse.

### Motor Cortex Forelimb Representation is Composed of Joint-specific Subregions

**Figure 2:**
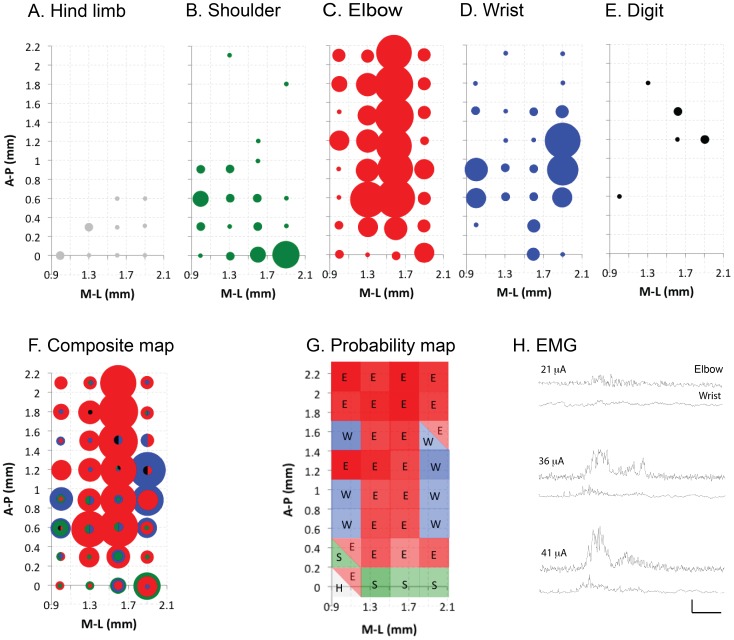
Joint-based organization of the forelimb region of the mouse motor cortex. Frequency distribution plots (A–E) show response topography. Axes show distances from bregma of the motor cortex area mapped. Medial-lateral (M–L) and anterior-posterior distances mapped were up to 1.9 and 2.1 mm respectively from bregma, which corresponds to most of the forelimb representation. Circle diameter for responses is directly proportional to response frequency across animals (10 responses maximum). (F) Representation maps were overlaid to determine any joint-bias within the cortical representation in the composite map. (G) The frequency plots were converted to an overall probability map based on the relative frequency of a dominant response at a specific site in relation to all responses at that site. Map shows the locations of dominant hind limb (H, grey), shoulder (S, green), elbow (E, red) and wrist (W, blue) responses. Shade intensity is directly proportional to the probability of provoking a response for a particular joint. (H) Representative EMG data from contralateral forelimb muscles in response to: threshold cortical stimulation for evoking an elbow response (21 µA, top traces); threshold cortical stimulation for evoking a wrist response (36 mA suprathreshold cortical stimulation for evoking both elbow and shoulder responses (41µA, bottom traces). Vertical and horizontal scales are 0.5 V and 20 ms respectively.

ICMS evoked movement about the shoulder, elbow, wrist, and digit joints (n = 35 mice), as other studies have shown in the mouse [25], [26]. Typically, ICMS at threshold evoked movement about a single joint. Movements about multiple forelimb joints were recruited at stimulus intensities that were markedly higher. On average, we needed to increase stimulation amplitude by 15 µA (approximately 1.7 times threshold) before a second joint was recruited. Single joint motion at threshold corresponded to activation of muscles acting at that joint, assessed using EMG recording. A representative pattern of EMG activation evoked at threshold from an elbow site is shown in Figure 2H. Threshold stimulation (21 µA) evoked elbow flexion. This was associated with a biceps, not a wrist, response (Figure 2H; top pair of traces). As the current was increased to 36 µA movement about the wrist occurred, which was associated with a wrist extensor EMG response (Figure 2H; bottom traces). Despite a multijoint response at higher currents, the response was dominated by the muscle/joint motion evoked at threshold.

Within the territory explored, we examined a total of 304 sites across all animals and forelimb responses were evoked at 273 of these sites (Figure 2B–E). In addition, we obtained 11 hind limb responses from the caudal region of the mapped area (Figure 2A). Some of these hind limb responses were evoked with either a forelimb shoulder response (2/3) or an elbow response (1/3); the remainder were hind limb only responses. The average threshold for evoking hind limb responses was 23±7 µA. No motor responses were evoked from the remaining 20 sites.

For the forelimb, responses evoked at the elbow were most common, followed by the wrist, shoulder, and digit. The maps in Figure 2B–E represent the locations and frequency (circle diameter proportional to frequency of occurrence across different animals) for evoking forelimb responses. Shoulder responses occurred 13.9% within the mapped area and they were located in the caudal half of the forelimb zone (B). Elbow responses were the most frequent (57.1%) and were represented throughout the mapped area (C). Stimulation at all of these coordinates evoked elbow flexion, although not at all sites in a single animal. Elbow extension was not observed. Wrist responses were the second most frequently observed (25.6%). Similar to the elbow, these responses were evoked from sites throughout the mapped area of motor cortex, with a bias for the medial and lateral margins of the mapped motor cortex forelimb area (D). Digit responses (E) were rare (3.3%), with many occurring with wrist responses at threshold.

The average threshold for the shoulder sites (15±2 µA) was significantly lower (one way ANOVA, P<0.05; DF = 3, F = 3.983, P = 0.018) than the elbow, wrist and digit, which were not different from each other (22±2 µA, 23±2 µA, 24±3 µA respectively). For a minority of responses (12.8%), which were without any motor cortex regional localization, it was not possible to distinguish among the multiple joint movements at threshold and these were termed multi-joint responses. The most common multi-joint responses involved the elbow with either of the other joints (68.6%). Overall, the average threshold of multi-joint responses was not significantly different from single joint responses (19±2 µA and 22±1 µA respectively, unpaired t-test, p>0.05).

We constructed a composite map (F) and a map of the probability of evoking a particular response from the composite map (G). The saturation of the color representing that joint is proportional to the probability for evoking the dominant response. At each of the 32 sites sampled, one joint was most likely to be evoked. Several shoulder- and wrist-dominant sites co-represented an additional joint. The elbow was the only joint that was solely represented at some coordinates (i.e., solid red). Although our findings indicate an underlying consistent somatotopy, they stress that mouse forelimb motor cortex is organized into regions where a given joint can be represented at multiple sites, as others have reported for monkey [27], [28], in a probabilistic manner. These regions likely evoke responses via specific descending CSTs that interact with spinal motor circuits. We next examined CST projections from identified joint specific zones.

### Corticospinal Segmental Terminations from Joint-specific Sites Target Different Zones at C7/C8 but not at C5/C6

**Figure 3:**
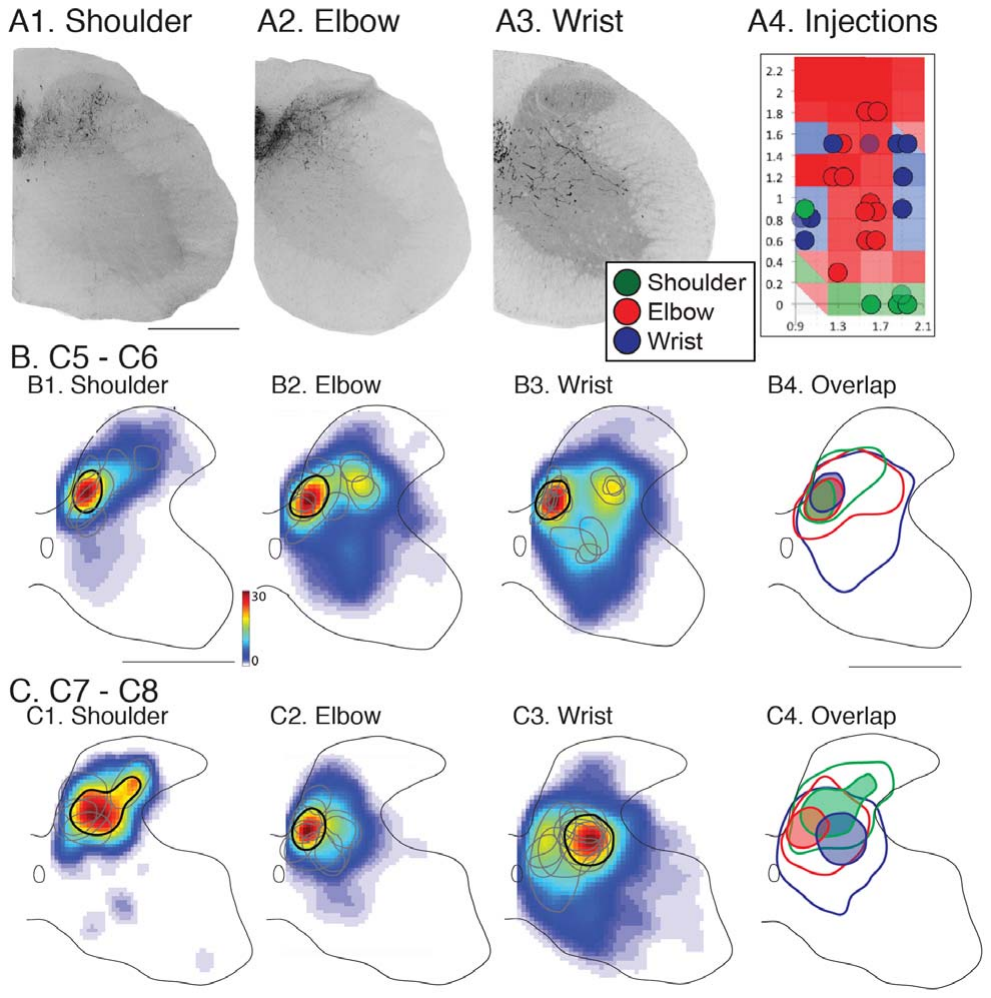
CST termination patterns from joint-specific motor cortex sites. Anterograde tracer injections were made into joint-specific sites in the motor cortex (A4; color code indicated in inset). Injection sites are shown overlaid on the joint probability map from Fig. 2G. Lightly shaded blue and green circles for wrist and shoulder respectively indicate injection sites where the dominant response and termination pattern were of the same group, yet was accompanied by a second smaller joint response at threshold. A1–3. Micrographs (inverted fluorescence images) of single sections showing CST labeling produced from injected (A1) shoulder, (A2) elbow, and (A3) wrist sites. B. Distribution of CST labeling at C5/C6. Average heatmaps for motor cortex shoulder elbow, and wrist sites (B1–B3). Black contours indicate the boundary of the high-density labeled region (≥60%) based on the averaged heatmap. Gray contours show high-density labeled region from each individual animal. B4 shows overlap of high-density (filled shapes) and low-density (10%; open shapes). Shading and line color according to the inset. C. Same as B, but for C7/C8. Color scale represents number of pixels per mm^2^. Scale bar = 500 µm.

To identify contralateral spinal termination patterns from motor cortex sites evoking movements of each of the forelimb joints, we injected anterograde tracers (BDA, LYDA or DAF) at sites whose principal joint actions were identified using ICMS (n = 23 sites; Figure 3A4; shoulder only: n = 4, shoulder>elbow; n = 1; elbow only: n = 11; wrist only: n = 7)). We examined the distribution of CST labeling in the C5/C6 segments and the C7/C8 segments. Retrogradely labeled deltoid, biceps, and wrist extensor motoneurons are present throughout these segments ( [22]; see below). Joint-specific sites projected consistently to a dense core region on individual spinal sections (typically a single site; occasionally 1 or 2 additional sites of much lesser density) within a more variable and broad sparsely labeled region (Fig. 3A1–3). In these representative examples from the C7/C8 levels, the shoulder, elbow, and wrist zones projected preferentially to the dorsal horn, medial dorsal horn and intermediate zone, and more broadly within intermediate zone and ventral horn, respectively. The densely labeled core regions were highly consistent between sections within animals. When we examined the distributions of CST terminal labeling from each motor cortex joint zone across all animals there were characteristic regional differences within C7/C8 but not in C5/C6.

For C5/C6 (B1–3), differences in the CST termination fields for motor cortex shoulder, elbow, and wrist joint zones were not apparent, as shown on the averaged color coded regional density plot (or “heat map”). The light gray lines enclose the region of densest labeling in each animal (60%; corresponding to orange in the color scale). The black line marks the average dense region based on the average heatmap. In all animals, the core of densest labeling for each joint was located in the medial dorsal horn-intermediate zone. These data are summarized in B4 (elbow, red; shoulder, green; wrist, blue), which shows overlap of both the dense zones (central filled profiles) and the sparsely labeled regions (10% density, corresponding approximately to gray-blue; larger, open profiles).

By contrast, there were clear differences in joint termination fields at the C7/C8 level (C1–3). CST shoulder site core terminations for all animals were restricted to laminae 4–5, as shown on the averaged color coded regional density plot (C1). M1 elbow sites (C2) terminated primarily in the medial portion of the deep laminae of dorsal horn and intermediate zone, ventromedial to the shoulder region. We noted for the elbow that more rostral sites in motor cortex had slightly more axons terminating ventrally in the spinal intermediate zone. The average antero-posterior bregma coordinate for this group (n = 4) was 1.5±0.2 and the remaining sites (n = 7), 0.8 mm ±0.1 mm (p<0.01, one way ANOVA). Core CST label from motor cortex wrist sites (C3) was preferentially located within the deeper dorsal horn laminae and the central intermediate zone (laminae 5–7) lateral to the elbow and ventrolateral to the shoulder regions. The region of sparser wrist labeling had the most ventral pattern of all joints. In some experiments we identified sparse projections from wrist sites into the region of the dorsolateral motor pools. In a subset of animals (n = 7), two different tracers labeled different motor cortex joint zones effectively. In these side-by-side comparisons, there were differences in the CST termination patterns for the two different joint zones. Figure S1 shows an example of wrist and shoulder zone labeling from the same animal. There was overlap at C5 and distinctive projections at C7. The pattern of overlap is summarized in C4; each core of joint labeling (filled shapes) occupied a distinct but partially overlapping territory. By contrast, the sparsely labeled regions (open shapes) showed more complete overlap.

**Figure 4:**
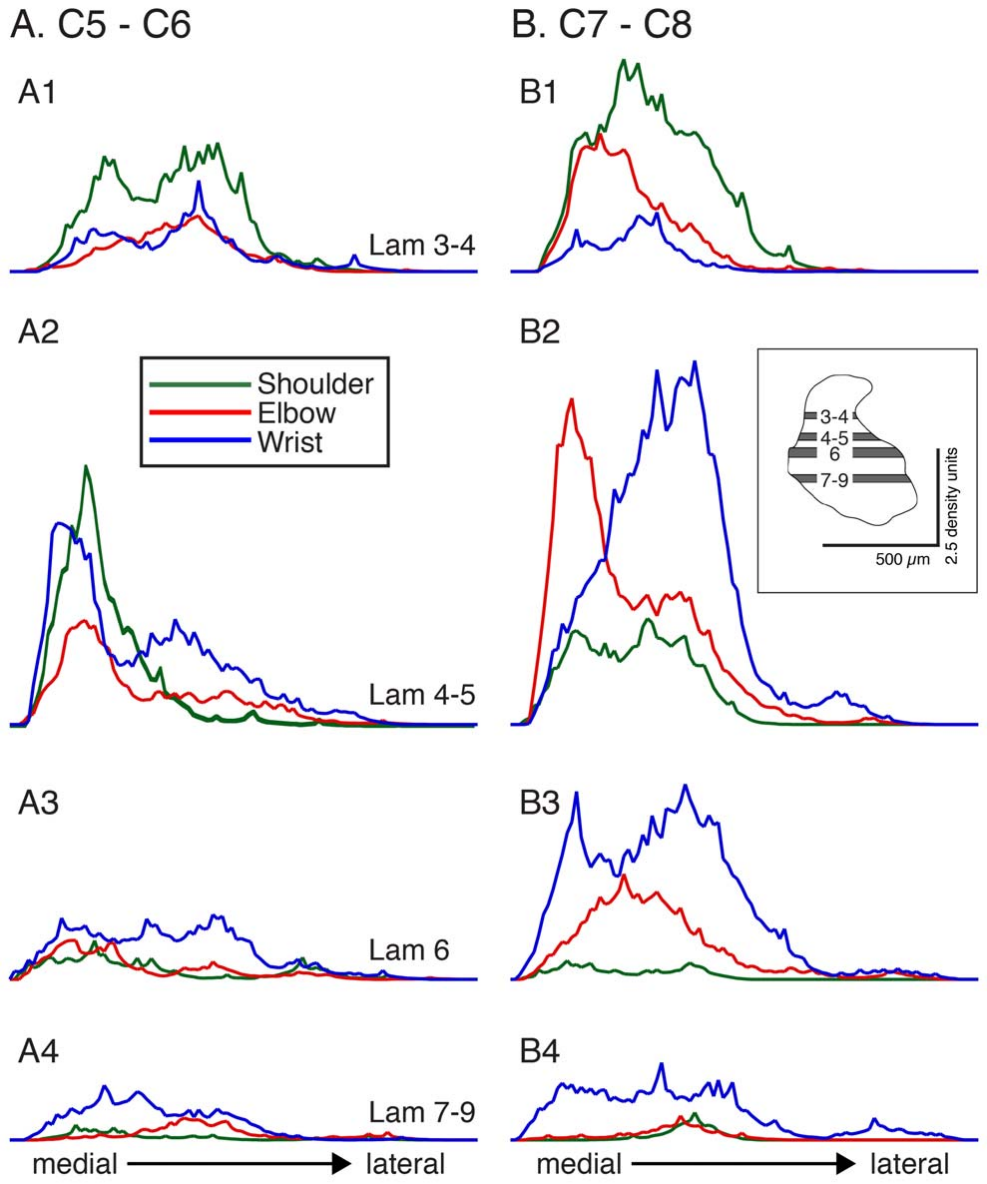
Density of CST anterograde label at 4 dorsoventral levels. A. C5/C6. B. C7/C8. Each row plots average label for ROI (inset, A2) from medial to lateral. Inset. Scale bars: 500 µm; 2.5 density units.

We conducted a laminar analysis to determine if these topographic differences in joint-specific projection patterns were significant across animals. We analyzed CST label density in 4 regions of interest (ROI, Fig. 4B2, inset). Each graph plots the total amount of labeling within the ROI, from medial to lateral, averaged across all animals for each joint group. At C5/C6 (Fig. 4A), CST terminations for each cortical joint zone largely overlapped. Note how peak shoulder, elbow, and wrist labeling overlapped in the medial portion of laminae 4–5 (A2). At C7/C8 (B), four key differences emerge. First, CST labeling was greater overall than at C5/C6. Second, shoulder zone projections to laminae 3–4 (B1, green) were denser than more ventral regions. Third, in laminae 4–5 (B2), peak elbow labeling (red) was located medial to wrist zone (blue) labeling. Fourth, in laminae 6 and 7–9 there was more labeling from M1 wrist zones than for the other joints (B3, B4). For C7/C8, a two way ANOVA revealed that the main effects of joint (DF = 2, F = 3.18; p = 0.047) and dorsoventral level (DF = 3, F = 4.88; p = 0.0039) were significant. Importantly, there was a significant interaction between joint and dorso-ventral ROI (F = 3.486; p = 0.005). For C5/C6, the main effects of joint (DF = 2, F = 4.054; p = 0.02) and dorsoventral level (DF = 3, F = 4.15; p = 0.009) were significant but, in contrast to C7/C8, there was no significant interaction between joint and dorso-ventral ROI (F = 0.635; NS). Whereas CST laminar differences are expected at both levels, given the distribution of CST terminations in many species, the presence of significant interactions between joint and laminae selectively at C7/C8 is surprising.

**Figure 5:**
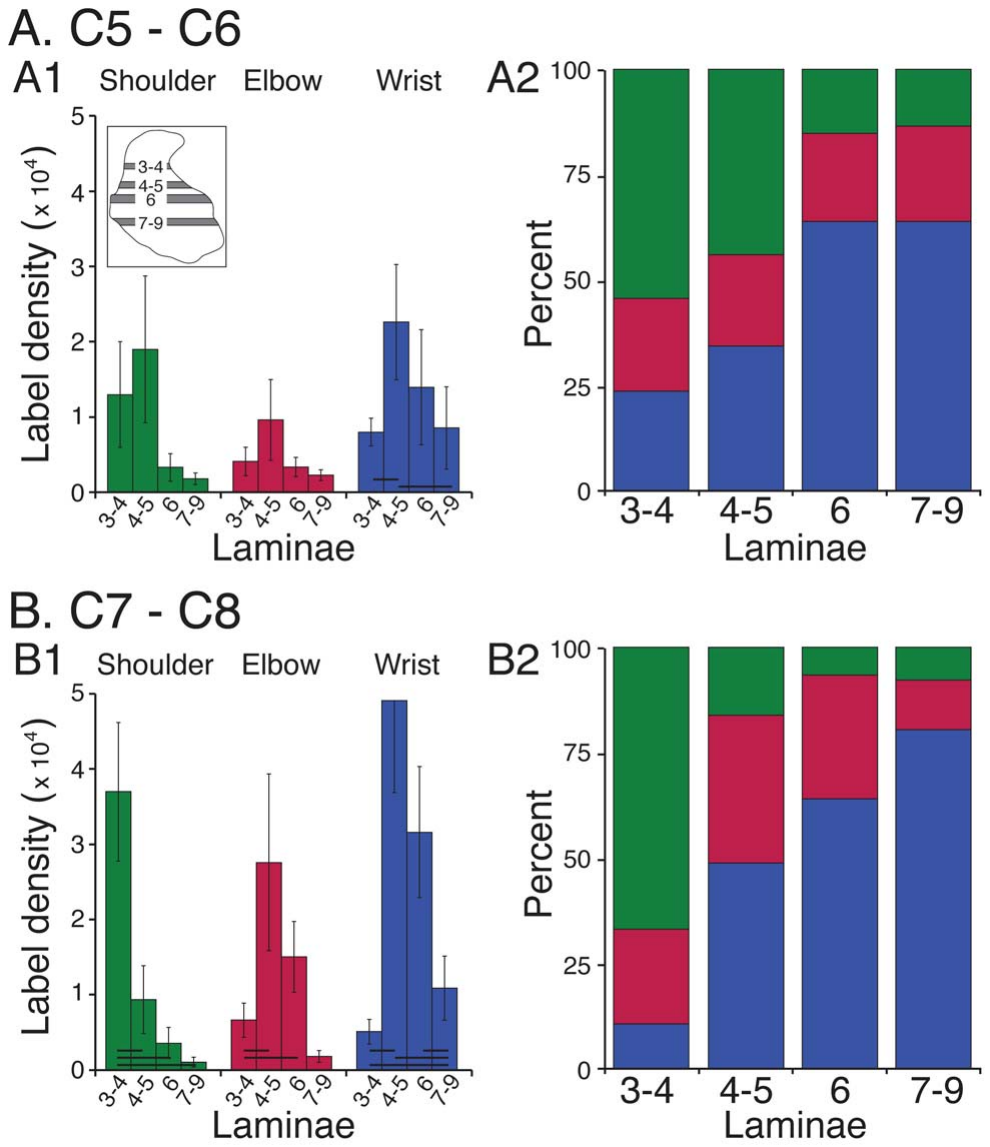
Dorsoventral changes in CST labeling. A. C5/C6. B. C7/C8. Left column plots mean ±SE of average label within each ROI (see inset), for each motor cortex joint zone. Bars plot values for laminae 3–4, laminae 4–5, lamina 6, and laminae 7–9. Horizontal lines at the bottom of each set of bar graphs indicate significant differences on post-hoc analysis (Scheffe test). Stacked percentage bar graphs in right column plot CST contributions from each motor cortex joint zone to the different dorsoventral laminar regions examined.

The topographic and ROI analyses described above point to potential important laminar termination differences for the motor cortex joint zones. To examine this further we plotted average label density for each joint zone across the 4 dorsoventral ROIs (Figure 5A1, B1). Most striking was the preferential projection of shoulder zones at C7/C8 to the dorsal portion of the dorsal horn and a progressive reduction ventrally (B1). Elbow and wrist at C7/C8 both projected maximally to intermediate laminae but, as shown in Fig. 4, the elbow projects medial to the wrist. Further, wrist zone projections at C7/C8 continued farther ventrally compared with shoulder and elbow zones. For C5/C6 (A1), there were minimal laminar distinctions. At C7/C8, there were significant differences in shoulder, elbow, and wrist zone labeling from dorsal to ventral (repeated measures ANOVA; shoulder: F = 9.35; p = 0.004; elbow: F = 4.38; p = 0.012; wrist: F = 9.208; p = 0.001). Post-hoc testing revealed that there also were significant across-laminar differences (shoulder: lam 3/4–4/5; 3/4–6; 3/4–7–9; elbow: lam 3/4–4/5; 3/4–7–9; wrist: lam 3/4–4/5; 3/4–6; 4/5–7–9; 6–7–9; Fig. 5). By contrast, for C5/C6 there was a significant difference in only wrist zone labeling from dorsal to ventral and a trend toward significance for the shoulder (repeated measures ANOVA; wrist: F = 3.84; p = 0.032; shoulder: F = 3.3; p = 0.071).

The complementary view to the differential laminar projections of the motor cortex joint zones is that each spinal laminar region collects CST inputs preferentially from one or another M1 joint zone: laminae 3/4 receive mostly shoulder input; laminae 4/5 elbow and wrist, but with wrist lateral to elbow; and laminae 6 and 7–9 receiving mostly wrist input. The stacked bar graphs (Fig. 5, A2 and B2) plot averaged label (mean of injection sites/animals for each joint and laminar region) as a percent of the total label within the laminar ROI. There was a decreasing gradient of labeling from dorsal to ventral for the shoulder and an increasing gradient for the wrist. This was most apparent for C7/C8. For example, at C7/C8 (B2) 60% of the label in laminae 3–4 came from M1 shoulder zones, 22% from elbow zones, and 8% from wrist zones. For the more ventral laminae, a flipped labeling pattern was observed with substantially more wrist than shoulder labeling. Elbow labeling was similar throughout, especially at C5/C6. Although this proportional pattern of labeling is based on a single measure of ensemble distributions, for C7/C8 it is a robust and significant pattern because it is based on significant dorsoventral within-joint distributions (B2). These data suggest that the different motor cortex joint sites have differential spinal terminations, especially to C7/C8, and further point to a more ventral pattern for distal joint control.

### Overlap between the Distributions of CTb Transganglionic Muscle Afferent Labeling and CST Labeling

**Figure 6:**
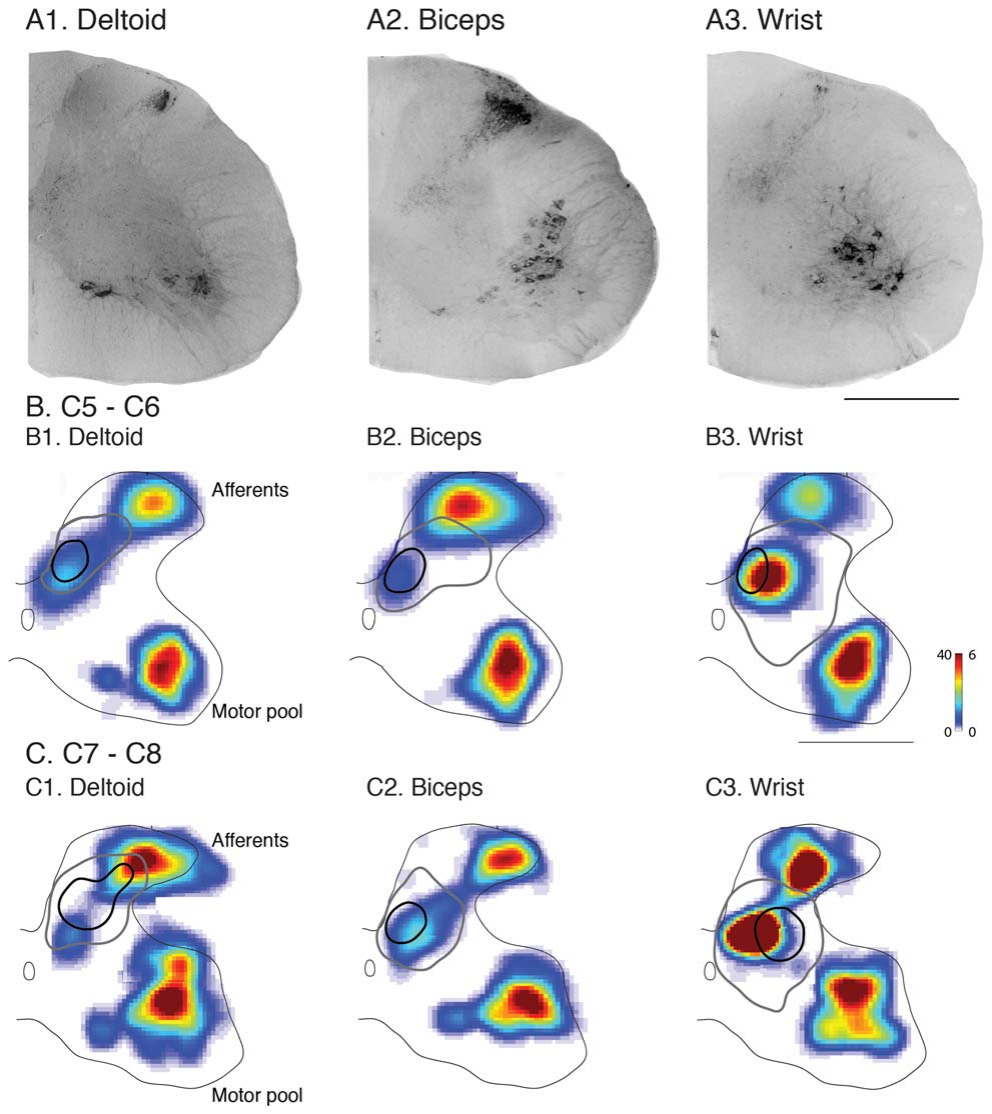
Cholera toxin b (CTb) labeling in the spinal cord after intramuscular injections and CST-interneuron topographic relationship. (A1–3) Examples of labeling with CTb subunit in one 40 µm section at level C7/C8, achieved after injection into either deltoids, biceps and the wrist extensor compartments. Matlab-generated heat maps of CTb-labeled spinal gray matter were produced from all sections for each muscle group at levels C5/C6 (B) and at levels C7/C8 (C). Heat maps show transganglionic labeling of the proprioceptive afferents in the dorsal and intermediate region of spinal cord (afferent; i.e. laminae 2–6) and retrograde labeling of the motoneurons in the ventral regions of the spinal cord grey matter (motor pools; i.e. laminae 9). Spinal overlap of motor cortex CST joint zones and proprioceptive afferent terminations are also shown in B and C. As in Figure 3, CST termination contours were set at 60% threshold (black contour) and 10% threshold (gray contour). The color bar demonstrates density of labeling as pixels per mm^2^ for proprioceptive afferents (left axis) and cells per mm^2^ for motor pools (right axis). Scale bar is 500 µm.

We next determined if the differentiated termination patterns of CST labeling at C7/C8, and the similar patterns at C5/C6, were associated with differences in motoneuron location or particular muscle afferent terminations (Figure 6). We used retrograde and anterograde transganglionic transport of cholera toxin B subfragment (CTb), from intramuscular injection, to identify the motor pool levels and the levels within which afferents from injected muscles terminate. For each muscle group injected–deltoids, biceps, and wrist extensors compartments (n = 3–5 sections, from 3 animals per group)–we found that CTb labeled motoneurons preferentially and extensively within the entire C5–C8 segment region (Figure 6; ventral horn labeling). This accords with recent findings in the mouse [22]. Whereas there may be more subtle quantitative differences between the numbers of motoneurons in C5/C6 versus C7/C8, the presence of robust motor pools at both levels does not explain the qualitative differences in CST termination patterns.

Muscle afferents transganglionically-labeled with CTb terminated within two distinctive regions, one within laminae 2–4 of the dorsal horn and the other within laminae 5–6 (Figure 6A1–A3; representative C7/C8 sections). Density maps (rows B, C) show that the dorsal fields were shifted somewhat for each muscle, suggestive of somatotopy, but that the intermediate zone afferent fields overlapped extensively. The average core CST terminations and more extensive sparse regions are superimposed on the CTb density heat maps (rows 2, 3). For the shoulder, at C5/C6 levels (B1) the CST overlaps with the intermediate zone-projecting proprioceptive afferents. At C7/C8 levels (C1), the shoulder CST projection targets a dorsal horn region that largely straddles the two proprioceptive afferent termination fields (C1). For elbow CSTs at all cervical levels (C5–C8), the high-density contour overlapped with that of the intermediate zone proprioceptive afferent fields of the biceps (B2–C2). For wrist CST, at C5/C6 the high-density CST terminations overlapped the proprioceptive afferents terminating in the intermediate zone (B3); at C7/C8 there was partial overlap between the high-density CST terminations and the intermediate zone afferent termination field (C3). We conclude that the subtle differences in the muscle afferent termination patterns from muscles acting at different joints did not differentiate the CST joint specific projections.

### High-density Wrist, but not Elbow or Shoulder, CST Termination Zones Target Territory of Last-order Interneurons at C7/C8

To further understand the functional significance of joint-specific CST segmental projections, we next determined the spatial relationship between CST spinal termination zones and the distributions of last-order (i.e., premotor) interneurons. Our starting hypothesis was that the different motor cortex joint-specific zones each target the spinal region containing the last-order interneurons that synapse on motoneurons innervating muscles acting on the represented joint. To identify last-order interneurons, we used retrograde transneuronal transport of pseudorabies virus (PRV) following intramuscular viral injection [14], [17]. Because PRV transport is not restricted to a single synapse, we first conducted a series of experiments (n = 15 mice) to determine the survival time required to label this neuronal population in adult mouse cervical spinal cord.

A post-injection survival period of 36 hrs did not result in viral labeling of any cervical neurons. Monosynaptic transfer across the neuromuscular junction, from muscle to the motoneurons, began to occur by 48 hr after the viral injections. At this survival time, only motoneurons were labeled. At 56 hr survival we observed very sparse labeling of interneurons, in addition to motoneuronal labeling. Sixty-four hours was the first survival time with substantial interneuronal labeling and, importantly, no label in motor cortex. This is consistent with the finding that monosynaptic connections between CST and motoneurons are sparse [29] or absent [30] in the rodent. At 72 hr, there was diffuse labeling of spinal interneurons throughout the gray matter and labeling in layer 5 pyramidal neurons of motor cortex [30]. This indicates transport beyond last-order interneurons at 72 hours. We therefore chose to use a survival time of 64 hr as selective for labeling putative last-order interneurons. It is bracketed by motoneuronal only transport at 48 hr and trisynaptic (from muscle) labeling at 72 hr. Thus, PRV can be used to label putative last-order interneurons in mature cervical spinal cord. Figure S2 shows the similar location of CTB-labeled biceps motoneurons and PRV-labeled biceps motoneurons in the ventral horn of the same animal.

**Figure 7:**
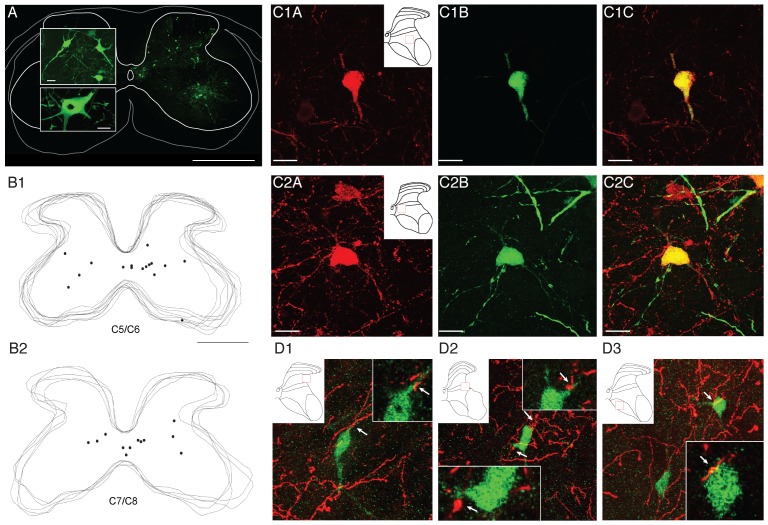
Spinal labeling of last-order (premotor) interneurons with PRV, co-labeling with ChAT, and contacts with CSTs. (A) Example of labeling in one 40 µm section achieved at 64 hours after intramuscular PRV injection. Large panel shows labeling on ipsilateral side (Calibration: 500 µm). There was minimal contralateral labeling. Top inset shows an example of labeled interneurons (located in lamina 4 on the section shown), and lower panel, a motoneuron, at higher magnification (Calibration: 25 µm). (B1–B2) Overlaid section images for two representative animals processed in Neurolucida showing positions of individual last-order interneurons from PRV injected into the deltoids, biceps and wrist extensors at levels C5/C6 (B1) and C7/C8 (B2) that also label positively for ChAT. (C1–C2) Confocal images of two representative PRV-ChAT double-labeled interneurons at levels C7/C8 (C1) and C5/C6 (C2). ChAT = red; PRV = green. (D1–3) (D) Confocal images of PRV-labeled interneurons receiving contacts from BDA-labeled CST axons terminals. PRV was injected into the biceps and wrist extensor compartments. Each panel shows a projection image (center, large image) and representative 1 µm optical slices (insets). Arrows show sites of contact,. Scale bars for A, large panel = 500 µm, smaller panels = 50 µm; B, same as A; C and D = 20 µm.

**Figure 8:**
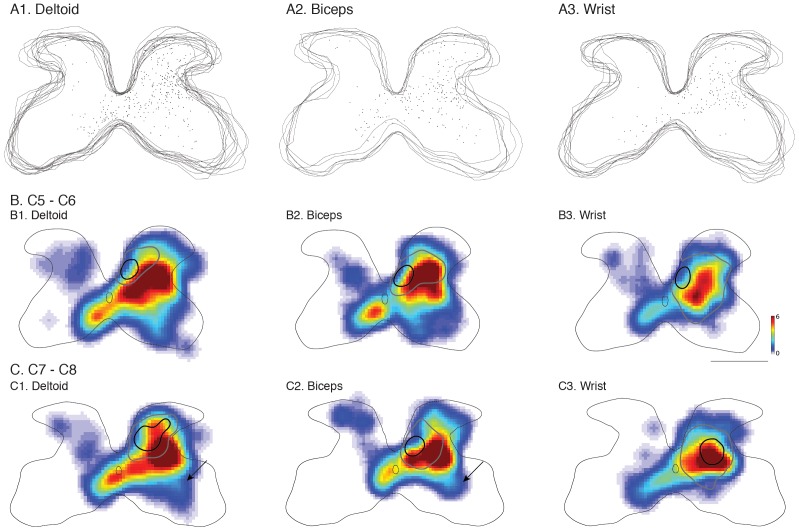
Segmental locations of last-order interneurons after PRV injections into individual muscle groups and CST-interneuron topographic relationship. (A1–A3) Overlaid C7–C8 section images processed in Neurolucida showing positions of individual last-order interneurons from PRV injected into the deltoids, biceps and the wrist extensor compartments in representative animals. B1–B3. Density heat map produced from all labeled sections at cervical levels C5–C6. C1–C3. Density heat map produced from all labeled sections at cervical levels C7–C8. Overlaid onto heat maps are shoulder, elbow and wrist high-density (black) and low-density (gray) CST termination contours. Color scale represents cells per mm^2^. Size bar = 500 µm.

Figure 7A shows representative spinal labeling after intramuscular PRV injections (combined deltoids and trapezius muscle compartments). The insets show the typical morphology of labeled interneurons (top) and a motoneuron (bottom). The location of the highest density of PRV-labeled interneurons (see Fig. 8) corresponds to that of several other methods for identifying last-order spinal interneurons, including dI3 premotor interneurons [31], and interneurons identified using a method that restricts transneuronal transport to only one CNS synapse in the neonatal mouse [13]. Further, we also identified a population of PRV-positive interneurons that immunostain for ChAT in lamina 10 (Fig. 7B,C). The location of these interneurons is similar to that of Pitx2 cholinergic interneurons in their cervical distributions [32] that make C-bouton contacts on motoneurons [33]. The bilateral distribution of these cholinergic interneurons is like that of lamina 10 cholinergic last-order interneurons in mouse pups [13]. These findings demonstrate that PRV labels similar interneuron populations as other approaches for identifying last-order spinal interneurons in rodents. Additionally, we found bouton-like contacts between labeled CST axon terminations and PRV labeled interneurons (Fig. 7D).

To determine shoulder, elbow, and wrist premotor interneurons, we injected PRV into the deltoid (n = 5), biceps (n = 4), and wrist extensors (n = 4) muscle compartments. Bartha strains 152 and 614 (expressing green and red fluorescent protein respectively) were injected into muscle pairs (biceps and wrist extensors; biceps and deltoids or deltoids and wrist extensors). We sampled a total of 57 sections for cervical levels C5/C6 and 46 sections for cervical levels C7 and C8 (3 sections minimum per animal at both levels). PRV-labeled interneurons associated with muscles at each joint were distributed throughout the ipsilateral gray matter and ventromedial contralateral gray matter (Figure 8, row 1; representative examples C7/C8). Color density plots (Fig. 8, rows 2, 3) pool data across all animals. The topographic distribution of PRV-positive interneurons was similar for muscles acting at each joint. There was a large, centrally-located, population of ipsilateral interneurons and several distinct populations of contralateral interneurons. Contralateral-labeled cells were consistent for deltoid and biceps, but not wrist. Interestingly, we also observed an ipsilaterally-labeled ventral group, which might be Renshaw cells [34], at C5/C6 (Fig. 8; B1, B2). Across all injections and animals, the majority of interneurons were labeled by PRV injection into a single muscle. The number of double-labeled interneurons (i.e., expressing both green and red fluorescent protein, indicating infection from muscles acting at two joints) was 21±4% for the side ipsilateral to the injections and 18±7% to the side contralateral to the injections.

For C5/C6, the densest CST termination zones (indicated by contours in Fig. 8) were all located medial/dorsomedial to the focus of densest last order interneurons (B1–B3). Substantial CST overlap with last-order interneuron territories occurred within the low-density CST fields. In the C7/C8 segments, the densest CST termination fields for both the shoulder and elbow (C1, 2) CST were primarily located medial/dorsomedial to the densest region containing the last-order interneurons. By contrast, the core wrist termination zone (C3) was co-extensive with to the densest region of last-order interneurons for wrist muscle. These findings show topographic co-registration between core CST wrist projections to PRV labeled interneurons at C7/C8. The other core CST termination patterns at C7/C8 and C5/C6 were shifted dorsomedially from the peak interneuron population.

## Discussion

The presence of the motor homunculus in the human, and similar cortical body representations across a wide range of species, shows that the motor cortex represents access to subcortical joint control circuits. This cortical motor representation is implemented, in part, by the CST. We propose a neuroanatomical basis for CST forelimb joint control at the spinal interneuronal level. We found that the motor cortex joint representations have differential spinal cord termination fields within C7/C8. Given the small dendritic fields of many spinal interneurons [11], [35], convergence between the high-density projections of different joint-specific cortical sites should be minimal. In contrast, substantial overlap between joint zones occurred in the regions of sparse terminations. These sparse overlap zones are where postsynaptic convergence between different CST joint-specific projections could occur. However, since the density of terminations is much lower in these zones, CST axons are likely to be much less effective in activating interneurons in these regions than CST axons projecting to the dense regions.

Our findings demonstrate a fractionated CST projection at C7/C8 compared to C5/C6. What function might the differentiated projection to C7/C8 confer? We found evidence that CST joint-specific projections are distinguished by terminations within the territories of putative last-order interneurons. We propose that cortical wrist sites, which projected preferentially to the focus of putative last-order wrist interneurons, comprise a more direct path to motoneurons than for shoulder sites, which project both within and outside the field of premotor interneurons. Surprisingly, elbow sites seemed to skirt the biceps premotor field. The differential CST terminatons at C7/C8 suggest access to different spinal interneuron circits for the various joints. By contrast, at C5/C6 focally dense CST projections from the different motor cortex joint sites overlapped highly. CST projections to C7/C8 are better suited for fractionated joint control; whereas projections to C5/C6, for a more integrated and co-active control. Surprisingly, at C5/C6 (and more caudal for the elbow sites) the dense joint-specific projections spared the territories of putative premotor interneurons. Importantly, this rostrocaudal difference is not explained by gross differences in the location of motor pools. As we show, and as recently reported [22], the forelimb motor pools we studied extend from the upper cervical cord to the caudal enlargement. Nevertheless, we cannot rule out that subtle differences in motor pool organization are important in shaping CST termination patterns.

### High-density and Sparse CST Spinal Termination Fields

Our findings suggest two populations of CST termination, one dense (“core”), which differentially targeted particular segmental territories at C7/C8, and a second that is sparse and diffuse. Whereas we show that CST axons can contact premotor interneurons, our interpretation stresses that it is the population of densest CST terminations that provides the most effective disynaptic access to motoneurons. While a single CST axon branch may make only a few contacts with a premotor interneuron, when this branch is located within the densest CST termination field it could affect the spinal neuron’s excitability together with the large cohort of other CST axons projecting to the same territory. It is unlikely that the sparse CST projections to premotor interneuron fields will be as effective as those within the dense focus because they function in relative isolation. The function of CST connections in the sparse fields may depend on convergent signaling from multiple cortical sites, other descending pathways, or somatic afferent inputs; they may serve a more modulatory function. The core/dense projection is better suited for feed-forward drive.

Co-registration of the cortical motor map and injection sites reveals the basic motor cortex representations of joint and, as we now show, segmental termination patterns that could provide differential access to segmental interneuron populations. However, the cortical joint map is not somatotopically fixed across animals. The probability of evoking a particular joint movement at a given location was high, but not 100%. Furthermore, at most sites, another joint could be recruited at a higher current. Multijoint and more integrative effects may depend on stronger drive to the sparse segmental territories, which might come about with longer duration stimuli in mapping studies [3] and persistent or more complex patterns of activity during motor behavior [4].

### Differential Spinal Joint Control Mechanisms

The joint-specific CST projections to C7/C8 show a remarkable amount of specificity that could lead to fractionation of connections with particular last-order and higher-order spinal interneurons [36]. Afferent fibers terminate within the dorsal horn, where they establish a somatotopy that, at least for the hind leg, is partly organized in relation to limb withdrawal reflexes [37], [38]. The dorsal termination from motor cortex shoulder zones suggests that its cortical control modulates dorsal horn sensory networks. CST regulation of spinal reflex function is not typically thought of as mediating feed-forward control of movement. However, movement directional specificity could be achieved by a CST projection onto dorsal horn reflex circuits with particular stimulus-response direction relationships that are established by peripheral afferents and early experience [38].

Cortical distal limb control may tap preferentially into a network in the intermediate zone that has direct access to motoneurons through last-order interneurons. Cortical wrist zones, but not shoulder or elbow zones, preferentially project to the territory of spinal dI3 interneurons in the cervical cord [31]. These interneurons are implicated in distal limb control. Plausibly, wrist/digit CST projections to these interneurons are important for manipulative skills. Many PRV-labeled interneurons were ChAT immunoreactive. Cholinergic last-order Pitx2 interneurons [32], [39], through their muscarinic actions [33], may play a role in the task-specific CST regulation of muscle force [32]. In the cervical spinal cord, localization of Pitx2 interneurons overlaps CST distal zone terminations.

Surprisingly, motor cortex elbow sites had their densest projections targeted to a restricted medial cervical region. This is a conserved CST termination zone, present also in humans examined using degeneration techniques [40]. There is a paucity of putative last-order interneurons in this cervical location. This region may contain spinocerebellar neurons, as reported for the rat cervical cord [41], although most are located rostral to C5. For the hind limb, this medial region has been shown to integrate monosynaptic CST input with local GABAergic inhibition that could comprise an internal feedback loop to the cerebellum [42].

### Mouse Motor Cortex and Encoding of Simple and Complex Motor Actions

Although our focus is on spinal mechanisms, our findings help inform the organization of motor cortex in the context of forelimb control. The forelimb motor map in mouse motor cortex, like other species, contains a complete body joint/muscle map [25], [26]. We suspect that, compared with cat and monkey, the small size of the mouse motor cortex places constraints on the number of joint-specific columns; such that the resultant map is sparse. Our injection sites averaged 279 µm in diameter. With these injections, we achieved remarkably consistent spatial specificity in the spinal cord. Cortical joint-specific sites, with differential C7/C8 projections, have a width that is similar to clusters of layer 2/3 motor cortex neurons (∼200 µm) that show highly correlated discharge patterns in behaving mice [43]. The temporal and spatial distribution of active motor cortex neuron clusters during grooming and locomotion could reflect control of forelimb joint motions during these two behaviors.

### Motor Cortex Co-represents Joint Control and Access to Spinal Circuits

Our findings provide two new perspectives on the anatomical mechanisms underlying CST control of limb muscles and joints. First, it was not known, nor even suspected, that joint-specific sites in motor cortex would have CST projections with differential segmental terminations. This is a new finding that begins to provide an anatomical explanation for the cortical joint/limb segment map. Importantly, the finding also provides a plausible new model for “upper motor neuron” control by conferring access to particular spinal interneuron circuits for joint control, especially premotor interneurons for distal joints. Second, differential segmental CST termination patterns reveal an unsuspected organization in the spinal cord. The patterns are neither an imprint of premotor interneurons, nor a simple means for integrating cortical control signals with muscle-specific proprioceptive afferents. Rather, it likely reflects the complex logic of the underlying circuits in those particular regions of the cervical cord. Whereas this logic that has yet to be elucidated, two circuits come to mind. One mediates “motor primitives,” limb movements with end-point control elicited by focal activation of lumbar spinal circuits [44], [45], while a other, a directionally-tuned limb withdrawal network [38], alluded to above. We propose that the CST joint-specific projections are accessing parts of these segmental effector circuits, through topographically distinctive terminations.

There is a growing inventory of genetically-identified spinal interneuron subtypes in the mouse [16], some of which have identified movement control functions [31], [32]. As in spinal cord, a genetic diversity is emerging for cortical output neuron subtypes [15], [46]–[48]. A critical question is if there is a molecular fractionation of CST neuron subtypes to complement the differential spinal connection patterns of joint control zones, or if the joint-specific projections are shaped by early motor experience.

## Supporting Information

Figure S1 **Spinal terminations of wrist and shoulder zones of the same animal.** BDA was injected into a wrist zone (A) and Lucifer yellow dextran amine, into a shoulder zone (B) in motor cortex. The locations of the injection sites are shown in the inset (wrist, blue circle; shoulder, green circle). Scale, 500 µm.
(TIF)

Figure S2 **Biceps motor pool at C7/C8 labeled by retrograde transport of CTb and PRV in the same animal.** A. CTb was injected into biceps to mark the locations of biceps motoneurons (n = 8 sections). The motor pool is located ventrolaterally in lamina 10. B. PRV was injected into biceps several days later. Note that biceps motoneurons are also located within ventrolateral lamina 10.
(TIF)
