# Supplementary figures and images for: Differential Joint-Specific Corticospinal Tract Projections within the Cervical Enlargement
Source: PLoS One. 2013 Sep 18;8(9):e74454. doi: 10.1371/journal.pone.0074454 (PMC3776849; doi:10.1371/journal.pone.0074454)

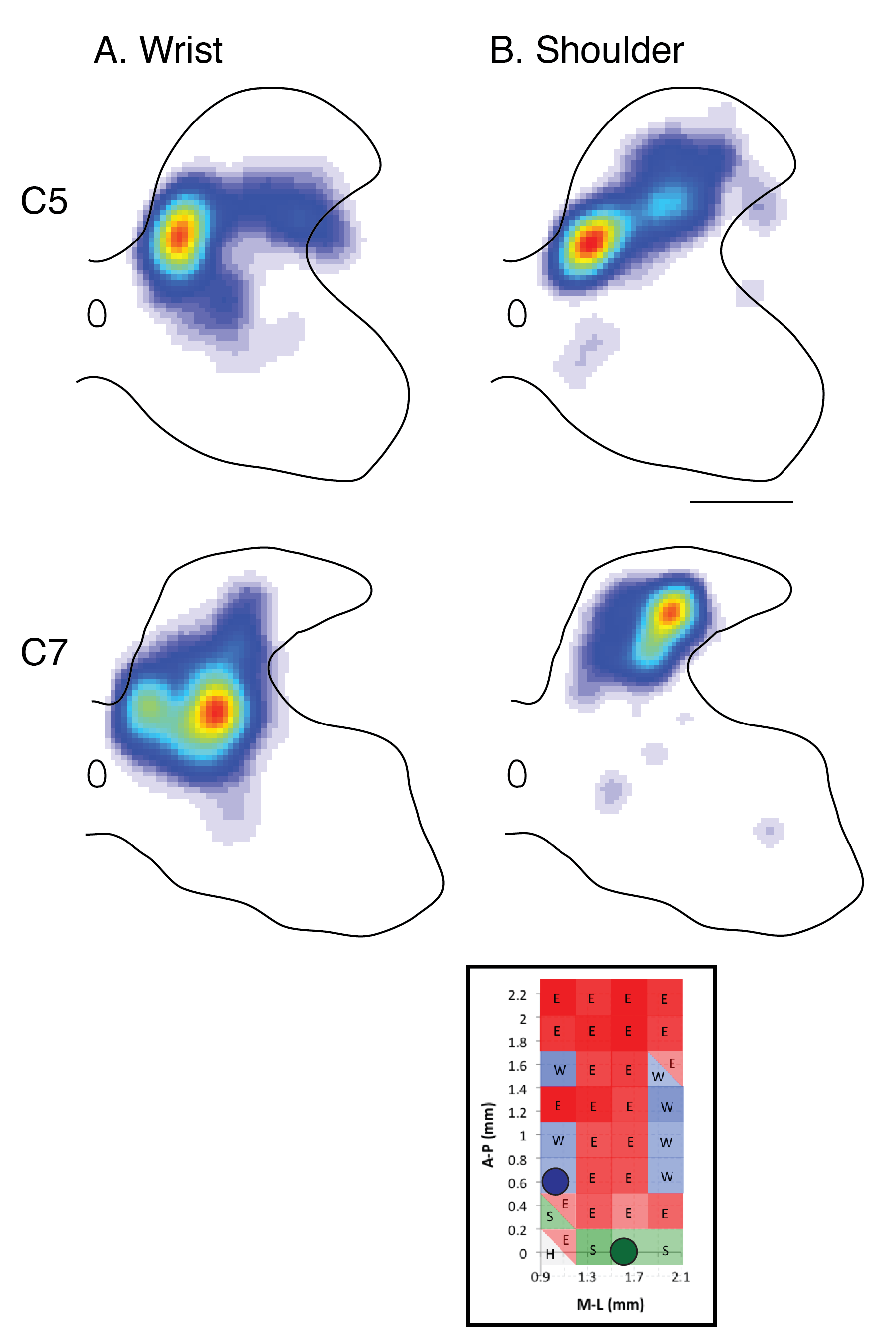

Supplement: Figure S1 — Spinal terminations of wrist and shoulder zones of the same animal. BDA was injected into a wrist zone (A) and Lucifer yellow dextran amine, into a shoulder zone (B) in motor cortex. The locations of the injection sites are shown in the inset (wrist, blue circle; shoulder, green circle). Scale, 500 µm. (TIF) [file pone.0074454.s001.tif]

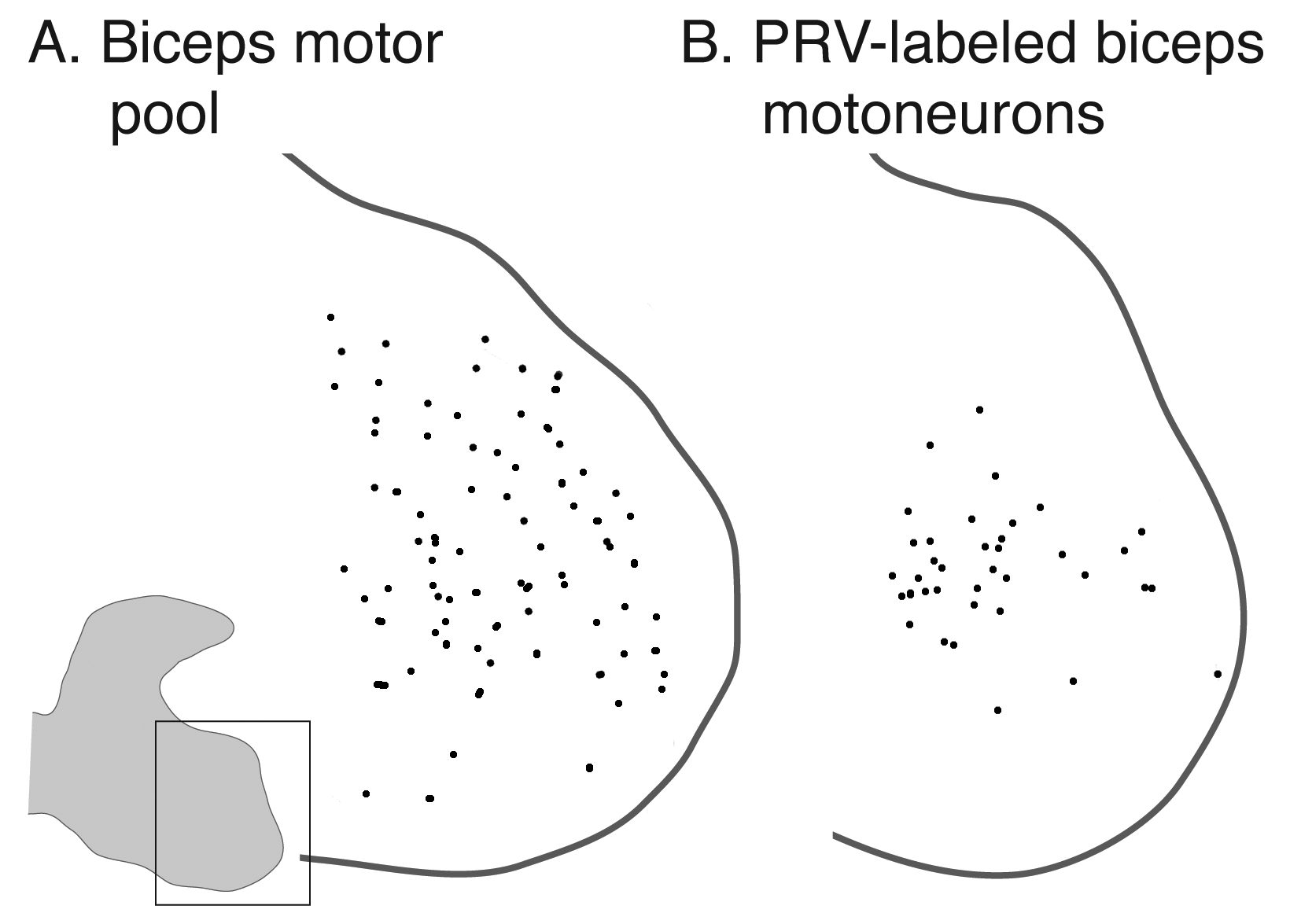

Supplement: Figure S2 — Biceps motor pool at C7/C8 labeled by retrograde transport of CTb and PRV in the same animal. A. CTb was injected into biceps to mark the locations of biceps motoneurons (n = 8 sections). The motor pool is located ventrolaterally in lamina 10. B. PRV was injected into biceps several days later. Note that biceps motoneurons are also located within ventrolateral lamina 10. (TIF) [file pone.0074454.s002.tif]
